# Combined Effects of Graded Foraminotomy and Annular Defect on Biomechanics after Percutaneous Endoscopic Lumbar Decompression: A Finite Element Study

**DOI:** 10.1155/2020/8820228

**Published:** 2020-08-25

**Authors:** Yefeng Zhang, Yan Li, Jingcai Xue, Yang Li, Guihua Yang, Guodong Wang, Tao Li, Junqin Wang

**Affiliations:** ^1^Spine Division of Orthopaedic Department, Shandong Provincial Hospital Affiliated to Shandong University, Jinan, Shandong 250000, China; ^2^Spine Division of Orthopaedic Department, The Second Affiliated Hospital of Shandong First Medical University, Taian, Shandong 271000, China; ^3^Department of Pediatrics, Taian Maternal and Child Health Hospital, Taian, Shandong 271000, China; ^4^Spine Division of Orthopaedic Department, The Second Affiliated Hospital of Shandong Traditional Chinese Medical University, Jinan, Shandong 250000, China; ^5^Department of Radiology, The Second Affiliated Hospital of Shandong First Medical University, Taian, Shandong 271000, China

## Abstract

Percutaneous endoscopic technology has been widely used in the treatment of lumbar disc stenosis and herniation. However, the quantitative influence of percutaneous endoscopic lumbar decompression on spinal biomechanics of the L5–S1 lumbosacral segment remains poorly understood. Hence, the objective of this study is to investigate the combined effects on the biomechanics of different grades of foraminotomy and annular defect for the L5–S1 segment. A 3D, nonlinear, detailed finite element model of L4–S1 was established and validated. Changes in biomechanical responses upon stimulation to the intact spine during different degrees of resection were analyzed. Measurements included intervertebral rotation, intradiscal pressure, and the strain of disc structure under flexion, extension, left/right lateral bending, and left/right axial rotation under pure bending moments and physiological loads. Compared with the intact model, under prefollower load, annular defect slightly decreased intervertebral rotation by −5.0% in extension and 2.2% in right axial rotation and significantly increased the mean strain of the exposed disc by 237.7% in all loading cases. For right axial rotation, unilateral total foraminotomy with an annular detect increased intervertebral rotation by 29.5% and intradiscal pressure by 57.6% under pure bending moment while the maximum corresponding values were 9.8% and 6.6% when the degree of foraminotomy was below 75%, respectively. These results indicate that percutaneous endoscopic lumbar foraminotomy highly maintains spinal stability, even if the effect of annular detect is taken into account, when the unilateral facet is not totally removed. Patients should avoid excessive extension and axial rotation after surgery on L5–S1. The postoperative open annular defect may substantially increase the risk of recurrent disc herniation.

## 1. Introduction

Approximately 80% of all adults are affected by low back pain (LBP) during their daily lives [1]. Lumbar spinal stenosis (LSS) is a common cause of LBP with leg pain and is often accompanied by lumbar disc herniation (LDH) [2]. The prevalent physiopathology of spinal stenosis is related to the compression of the nerve root by the herniated disc, hypertrophied facets, and ligamentum flavum [3]. According to the pathological zone, LSS can be classified into spinal central stenosis, lateral recess stenosis, and foraminal stenosis [4]. Although conventional open decompression surgical interventions are regarded as the gold standard treatment, minimally invasive technology has been widely used for LSS [5]. Percutaneous endoscopic decompression (PED) techniques are emerging minimally invasive alternative for treating LSS, which shows comparable clinical outcomes to the conventional open surgery with shorter operation times, lower levels of tissue trauma, and lower treatment costs [6, 7]. PED, including interlaminar PED, transforaminal PED, and percutaneous endoscopic lumbar foraminotomy (PELF), and the choice of the surgical method are primarily related to the type of LSS.

PELF via the extraforaminal approach is effective for the decompression of foraminal or extraforaminal stenosis [8]. Foraminal decompression is completed via the removal of hypertrophied bone tissues, thickened ligaments, and/or protruded disc fragments compressing the exiting nerve root [9]. To directly decompress the exiting nerve root and to ensure that the working space is wide enough for additional decompression, a unilateral superior facet is normally graded and removed from the outer surface of the facet joint using endoscopic burr and punches during PELF. Discectomy is optional during decompression surgery and performed when appropriate [5]. The postoperative annular detect (AD) is unavoidable in most cases after removing the extruded disc. Although more tissues can be preserved in minimally invasive surgery, the excision of the articular process and annulus fibrosus (AF) may still reduce spinal stability and alter the load-bearing and motion characteristics of the surgical and adjacent lumbar regions [10, 11]. Understanding the degree of spinal instability and the quantitative changes in the mechanical environment of the lumbar spine after PELF with discectomy is necessary for surgical optimization and postoperative prevention and treatment.

Postoperative evaluations have indicated the safety and effectiveness of the percutaneous endoscopic operation. However, the correlation between the extent of resection of posterior structures and the change of spinal biomechanics after PELF remains unclear, let alone the combined effects of the removal of facet and AF. The open AD as an iatrogenic trauma left after decompression/discectomy is classified as a surgeon-controlled risk factor for LBP recurrence [12]. Several groups have reported the individual biomechanical effect after the graded resecting articular process in the conventional open procedure. Zhou et al. [13] assessed five human cadaveric lumbar spines of the L4–L5 segment and observed that lumbar stability was significantly influenced after a more than 50% graded facetectomy. Natarajan et al. [14] reported that facetectomy with resection of more than 75% resulted in substantial changes in the rotation motion. Overall, higher grades of resection of the posterior structures were shown to be associated with a higher frequency of instability using the finite element (FE) method, while the impact of AD was seldom considered [15, 16]. Furthermore, the level of L4–L5 and superior levels are major objectives in previous researches. Due to the obstruction of the iliac crest and transverse processes, endoscopic surgery on the L5–S1 level is a challenge [9]. In terms of the L5–S1 level at which 75% of foraminal stenosis occurs, studies on decompression surgery are limited [17, 18].

The aim of the current study was to investigate the biomechanical effects on surgical segment L5–S1 and its adjacent L4–L5 segment, which contributed by different grades of foraminotomy and AD via PELF with discectomy. A 3D nonlinear FE model of L4–S1 lumbosacral spine was developed and validated. The changes in detailed biomechanical responses after each stage of resection were analyzed in terms of intervertebral rotation (IVR), intradiscal pressure (IDP), strains in the AF and nucleus pulposus (NP) for flexion, extension, left/right lateral bending (LB), and left/right axial rotation (AR) with or without precompressive follower load (FL).

## 2. Materials and Methods

### 2.1. Finite Element Model

**Figure 1:**
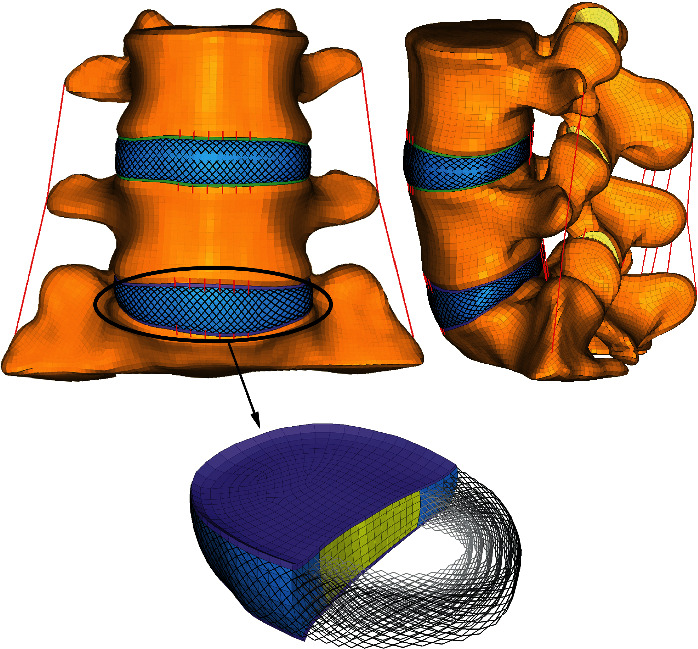
Finite element models of lumbosacral vertebrae L4–S1 and L5–S1 intervertebral disc.

A 3D nonlinear detailed FE model of L4 to S1 vertebra was established based on computed tomography (CT) images of a 36-year-old male volunteer with no spinal disease. The intact FE model consisted of 423987 elements and 136456 nodes. As shown in Figure 1, the FE model was asymmetric across the mid-sagittal plane and contained three vertebras, two intervertebral discs, and seven major ligaments.

In addition, the cancellous bone was modeled as a 4-node tetrahedral element, and other components including cortical bone, annulus ground substances, NP, cartilaginous endplate, and facet joint cartilage were meshed using 8-node hexahedral elements. The AF was assumed as a composite of the annulus ground substance reinforced by collagen fibers. The annulus collagen fibers and all ligaments (anterior longitudinal, posterior longitudinal, intertransverse, interspinous, supraspinous, ligamentum flavum, and capsular (CL)) were represented by nonlinear 1D truss elements. Six alternative layers of fibers were modeled. The collagen fibers that supported the AF matrix were angled at 30 to 45° with respect to the horizontal plane and varied from the inner to the outer lamina of the AF [19]. The facet joints had an initial gap of ∼0.5 mm [20] and consisted of two frictionless solid cartilage layers with a thickness of 1.25 mm [21].

### 2.2. Material Properties

Table 1 lists the material properties of all the spinal components of the FE model. Linearly elastic and transversely isotropic materials were used to simulate the mechanical behavior of cortical and cancellous bone, respectively [22, 23]. The material of the endplate was taken as homogeneous and linearly elastic [29]. The behavior of the joint facet cartilage was simulated by using Neo–Hookean, hyperelastic constitutive relation [24]. Ligaments were described by nonlinear functions based on previous reported stress-strain relationships and further calibrated data [26, 30]. The nonlinear response of the annulus ground substance was modeled as a hyperelastic Yeoh material law [25]. The fluid-like behavior of the NP was simulated as nearly incompressible and hyperelastic which is described by a Mooney–Rivlin formulation [28]. The mechanical behavior of the collagen fibers was represented by a 1D truss element with nonlinear function according to the previous protocols and the further calibrated values [26, 27]. Because the external lamellae were stiffer than the internal lamellae, the weight of fibers in different annulus layers was considered (innermost layer one: 0.65, layer two: 0.7, layer three: 0.75, layer four: 0.8, layer five: 0.9, and outermost layer six: 1.0) [31].

### 2.3. Graded Resection Models

**Figure 2:**
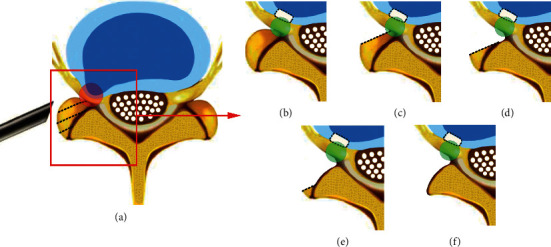
Schematic view of the graded decompression surgery of percutaneous endoscopic lumbar foraminotomy. (a) The nerve root was compressed by the facet and herniated disc in the red circle region. The facet, bulging disc, and free fragments can be removed by using a bevelled working sleeve. (b) The nerve root in the green circle region was exposed and decompressive after only the removal of the extruded disc (AUF-0). The postsurgical annular defect was left on the left posterolateral side of the annulus fibrosus and surrounded by a black dotted line. (c) The nerve root was exposed and decompressive after the removal of the extruded disc and 25% left superior articular process (AUF-25). (d) The extruded disc and 50% left superior articular process were removed (AUF-50). (e) The extruded disc and 75% left superior articular process were removed (AUF-75). (f) The extruded disc and total left superior articular process were removed (AUF-100). The dotted line on the superior articular process represents the dividing line of foraminotomy.

An iatrogenic-altered annulotomy hole left after the removal of the extruded disc in PED was developed on the left posterolateral part of AF for all surgical cases at the level L5–S1, which matches the size of the working cannula of about 6 mm width and 4 mm height [8]. According to the surgical procedure described in percutaneous endoscopic techniques [5, 32], different degrees of decompression studied at L5–S1 were simulated by the graded removal of the outer surface facet and the corresponding CL of the intact FE model (see Figure 2), as described hereinafter:

1. A single AD located at the left posterolateral part of AF with 6 mm width and 4 mm height was developed in all surgical cases
2. None (0%) left unilateral foraminotomy (AUF-0): the left superior articular process of S1 and the corresponding CL were preserved. Only the single AD was established
3. 25% left unilateral foraminotomy (AUF-25): the left superior articular process of S1 was removed by 25% with the corresponding CL
4. 50% left unilateral foraminotomy (AUF-50): half of the S1 left superior articular process was removed with the corresponding CL
5. 75% left unilateral foraminotomy (AUF-75): the left superior articular process of S1was removed by 75% with the corresponding CL
6. Total (100%) left unilateral foraminotomy (AUF-100): the total left superior articular process at S1 was removed with the corresponding CL

### 2.4. Boundary and Loading Conditions

In each model, the bottom layer of the S1 vertebral body was completely fixed in all six degrees of freedom. The load patterns were divided into two types: pure bending moments and bending moments with preloads. When calculating the influence under pure bending moments on the excisional models, a 7.5 Nm pure bending moment was applied to the top surface of the L4 vertebra in three main anatomical planes, namely, flexion, extension, left LB, right LB, left AR, and right AR. For simulating flexion, extension, LB, and AR in physiological conditions, an FL of 500 N was applied along the axial curve from L4 to S1 before the bending moment of 7.5 Nm was applied to the superior endplate of L4. The FL was applied using a connector element [26]. According to *in vitro* experiments, the cortical bones of adjacent vertebral bodies were attached by two pairs of connecter elements bilaterally. The FL directed to the centers of adjacent vertebral bodies in the sagittal plane. The connector elements acting on the motion segment were symmetrical on both sides. The input values of axial compressive forces were set as 250 N on each connector element. In total, twelve different loading situations were performed for the analyses of L4-S1 lumbar motion segments subjected to the decompression surgery.

### 2.5. Evaluation of Effects

The research objects of the influence of the mechanical response of the lumbar spine for PELD under different degrees of foraminotomy were the surgical segment L5-S1 and the adjacent segment L4–L5. The calculated values are detailed as follows: the IVR between the L5 and S1 vertebral body and L4 and L5, the IDP in both intervertebral discs L5–S1 and L4–L5, the strain of the AF in the L5–S1 disc and L4–L5 disc, and the strain of the NP (whole and adjacent to the AD) in the L5–S1 disc and L4–L5 disc.

All analyses were calculated using ABAQUS software (ver. 6.13-14, Dassault Systems, Versailles, France). The Full-Newton iteration method was used to solve the nonlinear equations.

## 3. Results

### 3.1. Validation

**Figure 3:**
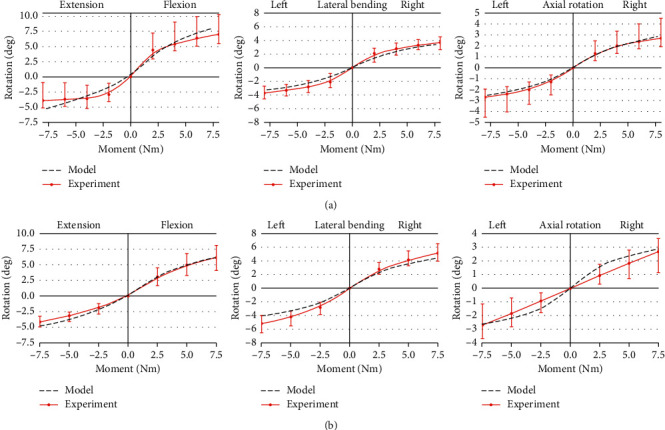
Comparison of the FE model from *in vitro* experiments [33, 34]. Rotation curves for (a) L5–S1 and (b) L4–L5 during flexion, extension, lateral bending, and axial rotation.

The IVR response of the L5–S1 and L4–L5 motion segments was consistent with *in vitro* experimental measurements [33, 34], as shown in Figure 3. The nonlinearity relationship between the moment and rotation was accurately simulated in the lumbar spine. In intact conditions of 7.5 Nm, the values were 8.03°, 5.19°, 3.15°, 3.37°, 2.48°, and 2.84° for L5–S1 under flexion/extension, left/right LB, and left/right AR, respectively. The corresponding values were 5.97°, 4.65°, 3.94°, 4.28°, 2.58°, and 2.85° for L4–L5.

**Figure 4:**
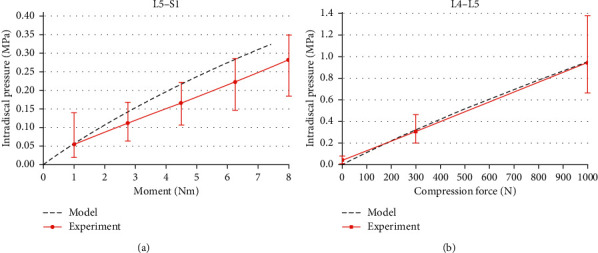
(a) Intradiscal pressure of the L5–S1 under extension moment. (b) Intradiscal pressure of the L4–L5 disc during compression.

The predicted IDP of the L5–S1 and L4–L5 discs in response to the extension moment loading and compression force fell within the range of available experimental data [35, 36], respectively. The IDP increased linearly with the applied moment and axial compression loading (Figure 4). The calculated IDP was 0.13, 0.24, and 0.32 MPa in extension for 2.5, 5, and 7.5 Nm bending moment for L5–S1 level, respectively. The values of predicted IDP in the disc L4–L5 were 0.31 MPa at 300 N compression and 0.94 MPa at 1000 N compression.

### 3.2. Intervertebral Rotation

The calculated IVR in each motion direction for the intact model and the graded foraminotomy models of L5–S1 is detailed in Table 2. For pure moment loading conditions, the percentage (%) increases of AD in the L5–S1 (AUF-0) relative to the intact model in angular rotation were 1.3%, 3.8%, 2.8%, 1.0%, 2.4%, and 2.2% for flexion, extension, left/right LB, and left/right AR, respectively. For flexion, the three graded foraminotomies of AUF-25, AUF-50, and AUF-75 had almost identical effects on the IVR of 2.7%, while AUF-100 increased the IVR by 3.6%. The same trend was observed for extension and left LB, which increased to 5.3% and 3.3%, respectively, for AUF-100. Foraminotomy had a minor effect on the right LB and left AR. The differences between AUF-0 and AUF-100 for the increased IVR were a maximum of 0.3%. The AUF-25, AUF-50, AUF-75, and AUF-100 gradually increased the right AR by 4.8%, 7.0%, 9.8%, and 29.5%, respectively.

Considering the pre-FL, the IVR between L5 and S1 changed by 0.7%, 5.0%, 2.9%, −0.9%, 2.9%, and −2.2% after AUF-0, respectively, for flexion, extension, left/right LB, and left/right AR (Table 2). In general, foraminotomy resulted in slight increases in the angular rotation under all physiologic loading modes, except for right AR. Compared with the intact model, AUF-100 increased IVR by 2.4% for flexion, 9.2% for extension, 4.7% for left LB, -0.8% for right LB, 3.0% for left AR, and 39.7% for right AR. The variation trends of IVR for the different resections under various loads were consistent with those under pure bending moments. In the majority of loading directions, AUF-25, AUF-50, and AUF-75 resulted in similar IVRs with differences of ≤1.8%. For right AR, removing 50% and 75% of the S1 left superior articular process increased the IVR by 1.7% and 4.6%, respectively.

### 3.3. Intradiscal Pressure

**Figure 5:**
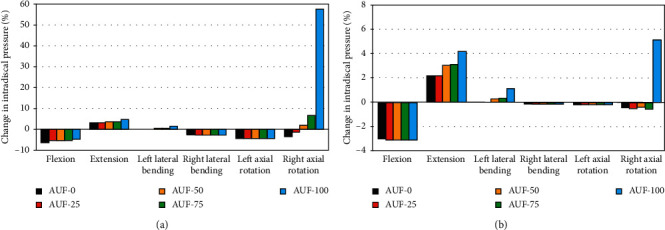
Change in intradiscal pressure (IDP) of L5–S1 intervertebral disc for different degrees of decompression. (a) Under pure moment bending. (b) Under preload bending.

For pure bending, partial annulus resection with graded foraminotomy reduced the IDP in most cases. After AUF-0, the percentage decreases of the IDP were 6.3%, −3.0%, 0.0%, 2.6%, 4.5%, and 3.5% for flexion, extension, left/right LB, and left/right AR, respectively (Figure 5(a)). Excluding right AR, total foraminotomy had almost no influence on the IDP. The difference between AUF-0 and AUF-100 when changing the IDP was a maximum of 1.8% for flexion, extension, and left LB, respectively, and 0.3% for right LB and left AR. For right AR, 25%, 50%, 75%, and total unilateral foraminotomy increased the IDP by −1.1%, 2.1%, 6.6%, and 57.6% respectively.

FL significantly increased IDP. For the same motion, the preload resulted in a loss of IDP changes under all loading modes on L5–S1, particularly during the right AR (Figure 5(b)). The AUF-50, AUF-75, and AUF-100 gradually changed the IDP of the right AR by −0.4%, −0.5% , and 5.1%, respectively. Similar IDP was observed under unilateral partial foraminotomy with differences ≤0.9% for the extension, and total unilateral foraminotomy increased the IDP by 4.2%, while for right LB and left AR cases, the maximum change of IDP was nearly zero compared with the intact model. In general, the changing trend was independent of the loading conditions and the extent of resection.

### 3.4. Strain in the Annulus Fibrosus

**Figure 6:**
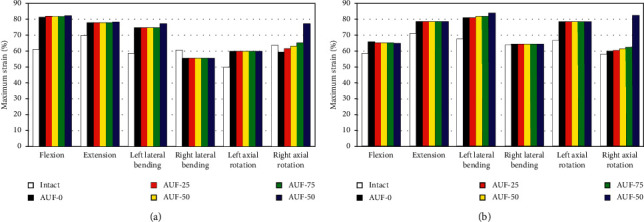
Maximum strain on L5–S1 annulus fibrosus (AF) for the intact model and different degrees of decompression. (a) The maximum strain on AF of L5–S1 under pure moment bending. (b) The maximum strain on AF of L5–S1 under preload bending.

Differences in AF strains between the intact spine and L5–S1 graded foraminotomy with AD are shown in Figure 6(a) for pure bending moments and Figure 6(b) in bending with a pre-FL. The maximum strains of AF during pure bending moments were 33.3%, 11.4%, 28.0%, −8.9%, 20.3%, and −7.1% higher after AUF-0 than in the intact spine, respectively, for flexion, extension, left/right LB, and left/right AR. Values of 12.7%, 11.0%, 20.3%, 0.5%, 17.7%, and 3.2% were observed for preload bending, respectively.

AUF-100 increased the maximum strain by 11.9% of pure bending, 10.7% of preload bending in extension, 32.5% and 24.5% in left LB, and 21.0% and 42.2% during right AR. However, unilateral foraminotomy procedures resulted in only minor differences for maximum strains in AF in comparison to the model with AD, excluding the right AR movement. AUF-25, AUF-50, and AUF-75 increased the strain in AF by −3.6%, −1.1%, and 2.1% for pure AR in addition to 3.9%, 5.7%, and 7.0% for preload AR, respectively. The peak strain was comparable to the annulus tissue surrounding the defect, except for right LB and AR.

### 3.5. Strains in the Nucleus Pulposus

Strains of the whole and exposed NP were calculated. The maximum strain of the entire NP for AUF-0 increased by 60.7%, 149.4%, 64.1%, 32.8%, 38.2%, and 13.0% relative to the intact disc for pure flexion, extension, left/right LB, and left/right AR, respectively. Under pre-FL, the values were 38.1%, 167.6%, 149.9%, −0.7%, 166.4%, and 91.5%, respectively. The strain distribution of NP was significantly influenced by the AD; after AUF-0, the peak strains of the whole NP appeared on the outer surface of the exposed NP under all loading conditions.

**Figure 7:**
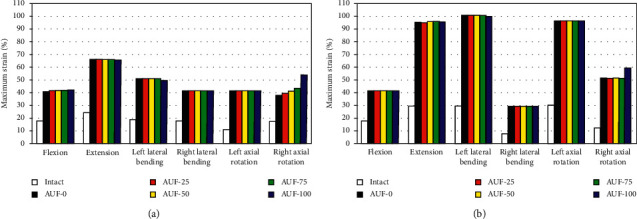
Maximum strain of the exposed nucleus pulposus (NP) in L5–S1 disc for intact model and different degrees of decompression. (a) The maximum strain of the exposed NP in L5–S1 disc under pure moment bending. (b) The maximum strain of the exposed NP in L5–S1 disc under preload bending.

The maximum strains of the exposed NP for AUF-0 were 128.4%, 171.4%, 169.7%, 136.6%, 288.1%, and 115.0% higher than those of the same NP region for the intact disc, respectively, for pure flexion, extension, left/right LB, and left/right AR (Figure 7(a)). Considering the preload, the increases were 130.8%, 222.1%, 247.2%, 278.5%, 220.3%, and 327.4%, respectively (Figure 7(b)). AUF-100 increased the strain by 206.7% of pure bending and 391.8% of pre-FL in right AR, respectively.

For right AR, the deformation was significantly influenced by graded unilateral foraminotomy surgery. Due to the extent of the enlarged resection, the strains, respectively, increased by 5.0%, 9.0%, 14.8%, and 42.7% for pure right AR. Total unilateral foraminotomy resulted in an increase of 15.1% under physiological AR. However, the increased strains only reached a maximum of 3.3% for pure extension and 0.7% for preload flexion.

### 3.6. Effects on Adjacent Spinal Segments

Different decompression procedures when performed under total unilateral foraminotomy had almost no effect on IVR, IDP, and disc strains in the adjacent L4–L5 segment under all loading conditions. The main contribution of the influence was caused by iatrogenic AD. The differences between different degrees of foraminotomy for the altered biomechanical behavior were a maximum of 0.1% in the adjacent segment.

## 4. Discussion

Numerous subjective clinical evaluations have shown that PED and discectomy are safe and effective methods for lateral recess and foraminal stenosis/herniation [5, 32, 37]. However, limited FE studies have been used to quantify the changes in spine biomechanical parameters following PELF with discectomy. Also, less research has been done on the L5–S1 spinal segment. Therefore, a 3D nonlinear detailed FE model for the lumbosacral spine L4–S1 was established based on CT data in the current study. Once the IVR and IDP of the model were validated through comparison with previous experiments [33–36], L5–S1 segment was modified to perform five degrees of resection to simulate the postoperative conditions of spinal decompression. The coeffect of graded PELF and AD on biomechanical response and spinal stability was investigated according to IVR, IDP, and the strain of disc under a pure bending moment or physiologic loads, quantitatively.

AD slightly affected the spinal deformation and IDP but resulted in a significant increase in the strain of disc structure. Despite the ideal minimally invasive discectomy, nerve roots were successfully decompressed only after the removal of the extruded disc, and free fragments without any resection of the articular processes can lead to iatrogenic damage to AF (AUF-0) [38]. The calculated IVR increased by an average of 2.4% for all simulations compared to the intact model. Relatively large changes occurred during extension, left LB, and AR. Furthermore, when the pre-FL was applied, the ROM of L5–S1 decreased in the right LB and right AR. These may be related to the weak area located at the left posterolateral side of the AF that reduced the compressive capacity of the disc in this weak area [39]. The IDP was minimally influenced by the AD, which decreased by an average of 3.3% for pure moments and 1.0% for preloads acting on the lumbar model, respectively. These differences can be interpreted as the IDP reached a high level under the FL, so the contribution of the bending moments acting on this pressure was reduced [40]. For all cases, the increase of mean maximum strain in the AF was 18.2% for pure bending and 9.3% under preload bending. Noteworthy, a remarkable increase of 168.2% for pure bending and 237.1% for preload bending was observed of the mean maximum strain in the exposed NP. In addition, the strain distribution of AF and NP was changed by the damaged AF, and the peak was found adjacent to the defect for all motions. Our findings indicated that the open AD seemed to become a channel for extruding the left NP and resulted in recurrent LDH and the need for further treatment. This is in agreement with the clinical outcomes that revealed that the AD is typically not treated and exacerbated during surgery and increases the risk of reherniation, ranging from 6% to 24% [12].

AD and unilateral partial foraminotomy (AUF-25, AUF-50, and AUF-75) together resulted in a minor biomechanical change in IVR of the L5–S1 segment. For all loading cases, the maximum increase always occurred during axial right rotation. The changing trend of calculated IVR was in agreement with the finding of Erbulut [15] stating that increased IVR for extension was 21.2% and 34.9% after 1/2 and 3/4 unilateral medial facetectomy. However, those were much higher than those calculated in the present study. And, the predicted results were more conservative than those of an *in vitro* investigation that concluded that the lumbar stability was unaffected when the degree of facetectomy did not exceed 50% [13]. The difference in the physiological structure of lumbar segments may contribute to the different results. Besides, in PELF, the initial target of the spinal needle was on the surface of the left superior facet of S1, and the outer surface of the S1 left superior facet was gradually excised for decompression, which preserved most of the facet surface. This totally differed from most studies, in which the medial section of the segment or the outer part of the inferior articular process was graded and removed [15]. As for IDP, it was significantly sensitive to the application of the FL. For the same motion, the IDP calculated with FL was approximately two times that without preload. The strains of AF and NP changed slightly with 25%∼75% resection especially when the FL was applied except under the right AR. Compared with the impact of AD, the maximum increase of strain in AF and NP was 10.0% and 14.8% which occurred in pure right AR after AUF-75, respectively.

Since unilateral facet and the surrounding capsular ligament were totally removed, 100% foraminotomy and AD significantly declined the spinal stability of the L5–S1 lumbosacral spine in the right AR and extension. And, the minor influence was found during flexion and LB. The increase of IVR after AUF-100 for the L5–S1 segment was 3.6%, 5.3%, 2.2%, and 16.1%, respectively, under pure flexion, extension, LB, and AR. Considering the pre-FL, the increases were 2.4%, 9.2%, 2.0%, and 21.4%, respectively. Similar observations were reported by Zeng et al. [41] in which the total unilateral facetectomy had a minor impact on the biomechanical responses in flexion and lateral bending but increases mobility by 51.6% in axial rotation. Rohlmann et al. [42] concluded that a left hemifacetectomy resulted in a substantial IVR change of about 32% in the rotation motion. Predicted results delivered the same changing trend between unilateral total foraminotomy and unilateral total facetectomy. However, the coeffect of resection that made the change of activity during extension cannot be ignored. Besides, even if we consider the effect of AD, unilateral total foraminotomy in PELF resulted in a relatively low increase in IVR of L5–S1 in torsion. An increase of IDP by 10.7% was recorded after the unilateral facetectomy in extension in an FE study [41], which was significantly higher than the predicted results of 0.9% in this study. Limited studies have documented the effect of facetectomy on disc strain. Total foraminotomy obviously increased the strain in the right AR. The maximum strains were all located close to the AD.

The AD and resection of the facet at L5–S1 had little to no influence on the IVR, IDP, and strain of the adjacent segments (L4–L5). These findings are in agreement with the findings of previous studies that reported only a negligible effect on the calculated parameters of the adjacent segments even when performed at two laminectomy levels [42]. Although direct biomechanical effects of the structural changes of the spine on adjacent segments are rare, these postoperative alterations in the intervened segment seem to be associated with further disc degeneration [43].

Although efforts have been made to improve the accuracy and reliability of the present FE simulations, including the simplification of the model geometrics, mesh refinements, calibration material parameters, and verification models, several limitations remain inevitable. Only a small AD (<6 mm width) with no NP resection was considered in this study. During surgery, the size of the defect and the removal of disc volume are dependent on the disease type and pathological stage. The postsurgical influences and complications are related to the size of AD [44]. In addition, these analyses did not consider changes in muscle forces that may be caused by the excision of the spinal structure during decompression surgery. To account for muscle force in the current model, the application of an FL is required, which is generated by the coactivity of the body weight and trunk muscle to simulate physiological loading conditions [26]. The effects of resecting the spinal structures may, however, differ for initial physiological biomechanical environments.

## 5. Conclusions

A nonlinear 3D FE model of L4–S1 lumbosacral spine was established, validated, and used to investigate the biomechanical effects of graded foraminotomy and AD on the L5–S1 segment. We concluded that spinal stability and pressure were largely unaffected by the AD but would lead to a remarkably increased risk of recurrent disc herniation from the biomechanical point of view. Partial foraminotomy performed in L5–S1 through PELF could effectively avoid spinal instability even if we considered the combined effect of AD. Excessive extension and AR were recommended to be limited for patients whose unilateral facet was completely removed. Besides, resection of the articular process or annular structures had almost no effect on the biomechanical behavior of the adjacent L4–L5 segment.

## Figures and Tables

**Table 1:** Material properties used in the present FE model.

Material	Young's modulus (MPa)	Poisson's ratio	Ref.
Cortical bone	10000	0.3	[22]
Cancellous bone	140/140/200	0.45/0.315/0.315	[23]
Joint facet cartilage	Neo–Hookean, *C*_10_=2, *D*=0.3		[24]
Annulus ground substance	Yeoh, *C*_10_=0.0146, *C*_20_=−0.0189, *C*_30_=0.041, *D*=0.3		[25]
Annulus fiber	Stress-strain curve/calibrated curve		[26, 27]
Nucleus pulposus	Mooney–Rivlin, *C*_1_=0.12, *C*_2_=0.03, *D*=0.3		[28]
Cortical endplate	1000	0.3	[29]
Ligaments	Stress-strain curves/calibrated curves		[26, 30]

**Table 2:** Intervertebral rotation of L5–S1 for the intact model and different degrees of decompression.

Motions	Loading	Intact	LF-0	LF-25	LF-50	LF-75	LF-100
Flexion (°)	Pure moment	8.03	8.13	8.24	8.24	8.25	8.32
	Pre-FL	8.17	8.22	8.30	8.30	8.30	8.37

Extension (°)	Pure moment	5.19	5.39	5.39	5.41	5.41	5.47
	Pre-FL	5.51	5.78	5.79	5.88	5.89	6.02

Left lateral bending (°)	Pure moment	3.15	3.24	3.24	3.24	3.24	3.25
	Pre-FL	2.86	2.94	2.94	2.96	2.96	2.99

Right lateral bending (°)	Pure moment	3.37	3.41	3.41	3.41	3.41	3.41
	Pre-FL	3.10	3.07	3.07	3.07	3.07	3.07

Left axial rotation (°)	Pure moment	2.48	2.54	2.55	2.55	2.55	2.55
	Pre-FL	1.88	1.93	1.93	1.93	1.93	1.93

Right axial rotation (°)	Pure moment	2.84	2.91	2.98	3.04	3.12	3.68
	Pre-FL	2.30	2.25	2.29	2.34	2.40	3.21

## Data Availability

The data used to support the findings of this study are available from the corresponding author upon request.
